# Injuries to the Eye

**Published:** 1907-11-02

**Authors:** 


### Ophthalmology.

### INJURIES TO THE EYE.

### I. FOREIGN BODIES.

In classifying injuries it is simplest perhaps to divide them roughly into three classes:—

1. Those due to foreign bodies.
2. Those due to penetrating wounds caused by sharp instruments.
3. Those caused by blows or contusions.

The first-class may be again sub-divided, as bodies may lodge on the surface of the cornea or in the conjunctival sac, or, on the other hand, may pierce the tunics of the eye and cause damage to the structures within the sclerotic.

The foreign bodies most frequently met with are minute particles of coal dust, emery powder, or pieces of metal. Any one of these lodging within the lids is apt to produce the symptoms of pain, watering, and redness of the eye, though occasionally foreign bodies may be found within the conjunctival sac which have remained unnoticed by the patient. It is noteworthy that the amount of pain and inflammation produced by a foreign body within the lids depends upon its position in relation to the cornea.

When examining the eye with a view to finding a foreign body place the patient in front of a good light and thoroughly inspect the surface of the cornea, direct the patient to move his eye about in every direction, and if it is not then visible evert the upper lid. If the foreign body be found embedded in the conjunctiva it may be possible to remove it with a small pledget of wool or lint, but if it has adhered to the cornea it will be necessary to use a local anæsthetic such as cocaine, grs. v. ad ℥j, before it can be successfully removed by a foreign-body needle or spud. A drop of this solution instilled into the eye and allowed to remain for five minutes is usually sufficient. The patient should be seated; the operator standing behind him should keep the lids apart with the thumb and index finger of the left hand, and holding in his right hand a sharp foreign-body needle, which has previously been sterilised, should insert its point under the foreign body and attempt to lift it out of its bed. Sometimes the offending grit is raised above the surface of the cornea, and a blunt-ended spud will be found sufficient for the removal, but all scraping and scratching of the cornea should be avoided in order to limit as far as possible the damage done to the corneal tissue.

If the foreign body is very deeply embedded in the cornea it may be necessary to scrape the superficial tissue away, as the point of the needle may not do more than push it between the layers of the corneal tissue; in such a case it is necessary to enlarge the hole in order to lift the particle out.

The urgency of removing a foreign body is due to the pain it causes, and also to the possibility of septic infection of the cornea. It must be acknowledged, however, that a septic foreign body is not likely to remain embedded for long, since by its very nature the ulceration around it will tend to loosen its hold on the corneal tissue, and it will fall out or be thrown off in the discharge. The practical point to be remembered is that it is never worth while inflicting extensive damage on the cornea in order to remove a foreign body, because it will probably detach itself in the course of a day or two, though it is essential to control any septic process, which might lead to a spreading ulcer.

After having removed the foreign body the treatment of the eye is that appropriate to a traumatic ulcer. The conjunctival sac should be thoroughly washed out with some warm boric lotion; should there be much inflammation atropine may be instilled, and if there is pain cocaine should also be used and a bandage applied.

A foreign body with sharp edges often pierces the cornea or sclerotic, and may be found lodged within the eye; if it can be seen in the anterior chamber an incision should be made in its immediate neighbourhood, and an attempt made to remove it with a pair of iris forceps.

After incision of the cornea the foreign body is sometimes carried outside the eye by the rush of aqueous as the knife is withdrawn, but if it is entangled in the iris it may be necessary to perform an iridectomy. If, however, the metal has entered the eye with force sufficient to carry it to the posterior part it may be embedded in the retina or choroid, and is sometimes impossible to see. Consequently any attempt to use an instrument in order to withdraw it would cause more damage than would be caused by the presence of the offending substance itself, and this is also true in cases where the foreign body can be actually seen. Under these circumstances the best method of extraction is by means of Haab's magnet. Skiagrams are frequently of value in locating the position of the fragment, and one should be taken before using the magnet. The eye, previously cleansed and anæsthetised with cocaine, should be brought near to the magnet, the lids being kept widely apart by a speculum. The electric current should then be started gradually; if the patient complains of pain in the eye it is taken as good evidence of the presence of a foreign body. Unless the metallic particle has become firmly embedded, the magnetic attraction will draw it forward into the anterior chamber, whence, if visible, it can be easily removed; should it, however, have become entangled in the iris or ciliary body, it will be necessary so to alter the direction of the magnetic pull by movements of the eye, as to entice the metal out of its entanglement and guide it through the pupil.

In some instances the foreign body remains in the lens, and if not visible on inspection a skiagram will probably reveal its presence and position; the treatment for this condition is the extraction of the lens through a linear incision.

If a foreign body will not yield to any of these methods of extraction, it must, of course, remain in the eye; and should panophthalmitis ensue the eye must be removed.

